# Sirtuin 5 regulates acute myeloid leukemia cell viability and apoptosis by succinylation modification of glycine decarboxylase

**DOI:** 10.1515/biol-2022-0832

**Published:** 2024-03-16

**Authors:** Jun Zhang, Cheng Luo, Haiying Long

**Affiliations:** Department of Hematology, The Second Affiliated Hospital of Guizhou Medical University, No. 3, Kangfu Road, Kaili, Guizhou, 556000, China

**Keywords:** SIRT5, succinylation, GLDC, acute myeloid leukemia

## Abstract

Acute myeloid leukemia (AML) is a blood system malignancy where sirtuin 5 (SIRT5) is abnormally expressed in AML cell lines. This study aimed to investigate the SIRT5 effects on the viability and apoptosis of AML cell lines. The mRNA and protein expression levels of succinylation regulatory enzyme in clinical samples and AML cell lines were detected by real-time quantitative polymerase chain reaction and western blotting while cell viability was measured using cell counting kit-8 assay. The apoptosis rate was assessed with flow cytometry. The interaction between SIRT5 and glycine decarboxylase (GLDC) was determined by co-immunoprecipitation and immunofluorescence staining techniques. Results indicated higher mRNA and protein expression levels of SIRT5 in clinical AML samples of AML than in normal subjects. Similarly, cell viability was inhibited, and apoptosis was promoted by downregulating SIRT5, in addition to inhibition of SIRT5-mediated GLDC succinylation. Moreover, rescue experiment results showed that GLDC reversed the effects of SIRT5 knockdown on cell viability and apoptosis. These results, in combination with SIRT5 and GLDC interactions, suggested that SIRT5 was involved in mediating AML development through GLDC succinylation. SIRT5 inhibits GLDC succinylation to promote viability and inhibit apoptosis of AML cells, suggesting that SIRT5 encourages the development of AML.

## Graphical abstract

**Figure:**
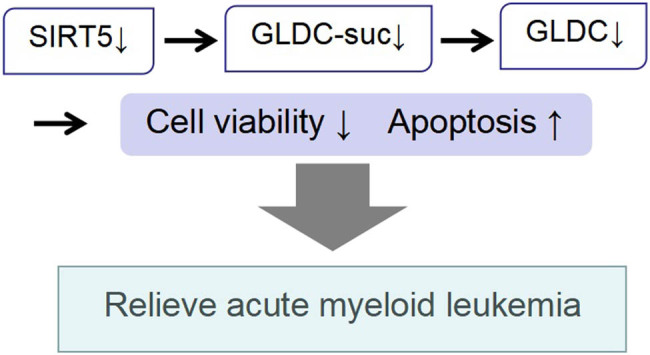

## 1 Introduction

Leukemia can be categorized based on immunological and morphological characteristics, cytogenic disorders, and different cell types into myeloid and lymphocytic, while acute and chronic leukemia is based on disease progression [1]. Acute myeloid leukemia (AML) is a malignancy of the human blood system and is a common form of acute leukemia, accounting for 80% of cases [2,3]. Its occurrence is independent of age and is characterized by abnormal self-renewal of leukocyte DNA and clonal expansion of leukocytes due to mutations in proliferation and differentiation [4]. The AML suffers a poor prognosis with less than 30% of a 5-year overall survival rate [5]. The available clinical treatment strategies include radiotherapy, chemotherapy, and surgery [6], which fail to ensure a complete cure due to associated high side effects and easy drug resistance emergence [7]. It is, thus, imperative to design a clinical treatment plan with targeted therapeutic outcomes per tumor pathogenesis [8]. Our study is therefore envisaged to improve AML prognosis and provide novel therapeutic targets to improve AML-targeted therapy.

Succinylation modification refers to the covalent binding of succinyl-coenzyme A (CoA) to protein lysine residues which is regarded as a novel post-translational modification [9] and widely exists in cells and participates in a variety of life activities [10]. Succinylation is also involved in metabolizing glucose, lipids, and amino acids via modulating gene expression and protease activity [11]. Several studies have shown that succinylation modification of substrate proteins or signaling pathways promotes or inhibits the progression of various cancers, such as thyroid, breast, gastric, and prostate [9,12–14]. However, the regulation pathway and mechanisms of succinylation modification in the occurrence and development of AML remain unknown.

Sirtuin 5 (SIRT5) is one of the desuccinylates [15], located within the mitochondria of cells, and belongs to the evolutionarily conserved Sirtuin family [16], and possesses unique enzymatic activity compared to other sirtuin family members. It has been reported to possess affinity toward negatively charged acyl groups, catalyzes deglutamylation and desuccinylation, and exhibits weak deacetylase activity [17], in addition to its neuro- and cardio-protective effects [18,19]. Moreover, SIRT5 activators or inhibitors could also be used clinically as effective drug targets for different cancers, such as liver, prostate, and breast [20–22]. It has been reported in a study that SIRT5 was abnormally expressed in AML cell lines [23]; however, the specific regulatory mechanism and the complete molecular route of SIRT5 in AML are not yet elucidated.

Glycine decarboxylase (GLDC), a mitochondrial pyridoxal 5ʹ-phosphate (PLP)-dependent enzyme, catalyzes the first step in glycine catabolism [24]. Abnormally expressed GLDC induces glycine accumulation, leading to neural tube defects and nonketotic hyperglycemia [25,26]. Recent studies demonstrated that GLDC was overexpressed in various cancers and plays a fundamental role in tumor growth, e.g., high levels of GLDC in non-small cell lung cancer-initiating cells are important for tumorigenesis through promoting pyrimidine biosynthesis, glycolysis, and sarcosine production [27]. Similarly, GLDC expression was significantly elevated in MYCN-amplified neuroblastomas, further promoting excessive cell proliferation [28]. However, the role of GLDC in AML has not been reported. Before conducting this study comprehensively, we conducted a preliminary experiment and found that SIRT5 can regulate the succinylation modification level of GLDC.

Therefore, this study aimed to investigate the specific SIRT5 mechanisms during AML progression. It is speculated that SIRT5 may be a pivotal regulator to mediate the viability and apoptosis of AML cells by regulating GLDC succinylation.

## 2 Materials and methods

### 2.1 Clinical sample collection

The blood samples were collected from 33 AML patients and 25 normal healthy volunteers. All patients were diagnosed with AML as per FAB and World Health Organization (WHO) typing criteria in our hospital. This study was approved by the Ethics Committee of our hospital and all participants signed informed consent before blood collection. Blood samples were handled strictly according to the *Declaration of Helsinki*.


**Informed consent:** Informed consent has been obtained from all individuals included in this study.
**Ethics approval:** The research related to human use has been complied with all the relevant national regulations, institutional policies and in accordance with the tenets of the Helsinki Declaration, and has been approved by the Ethics Committee of The Second Affiliated Hospital of Guizhou Medical University.

### 2.2 Culture of AML cells

AML cells including HL-60 and KG1a were purchased from ATCC (USA). HL-60 and KG1a cells were maintained with Dulbecco’s modified eagle medium (Gibco, NY, USA) containing 10% fetal bovine serum (Sigma, St Louis, MO, USA) and 1% penicillin/streptomycin (Sigma, St Louis, MO, USA). All cell lines were cultured in an incubator at 37°C with 5% CO_2_.

### 2.3 Cell transfection

Short hairpin RNA SIRT5 (sh-SIRT5), over-expression SIRT5 plasmid (SIRT5), over-expression GLDC plasmid (GLDC), and negative controls (sh-NC or vector) were synthesized in RiboBio (Guangzhou, China). When the fusion degree of HL-60 and KG1a cell lines was about 80%, Lipofectamine™ 2000 (Invitrogen, State of California, USA) was utilized to perform cell transfection. The cells were transfected with 100 ng shRNA and 30 μg over-expression plasmids. Forty-eight hours after transfection, transfected cells were collected.

### 2.4 Real-time quantitative PCR (qRT-PCR)

TRIzol reagent (Invitrogen, CA, USA) was used to extract total RNA from blood and cells. The BeyoRT™ III M-MLV (Beyotime, Shanghai, China) was used for RNA reverse transcription. PCR amplification was determined using the TB Green^®^ Premix Ex Taq™ II (Takara, Dalian, China) on the CFX96 TOUCH real-time PCR instrument (Bio-RAD, California, USA). mRNA expression was calculated using the 2^−△△Ct^ method. GAPDH was the internal standard. All primer sequences are available in Table 1.

**Table 1:** Primers used in our work

Name	Sequence (5ʹ–3′)
CPT1A-F	ATGCGCTACTCCCTGAAAGTG
CPT1A-R	GTGGCACGACTCATCTTGC
KAT2A-F	CTCTGCCTTAACTACTGGAAGC
KAT2A-R	GCCATCTGGTGTAATTGACCTTG
KAT3B-F	CGTTGCCCTATCTCCGTCTC
KAT3B-R	GGGAGCAATCGGGTAATTTTCC
SIRT5-F	TGGAGGAGGTTGACAGAGAGC
SIRT5-R	CTGCTGGGTACACCACAGA
SIRT7-F	AGAAGCGTTAGTGCTGCCG
SIRT7-R	GAGCCCGTCACAGTTCTGAG
GLDC-F	ATTTCTCGTTGATCCCCGTTG
GLDC-R	CACAGGGTAACTTCAGCTCAG
GAPDH-F	ATTGTTGCCATCAATGACCC
GAPDH-R	AGTAGAGGCAGGGATGATGT

### 2.5 Western blot

Modified western blot assay, as previously described, was used for determination of protein expression levels [29]. RIPA lysis buffer (Beyotime, Shanghai, China) was used to obtain total proteins from AML cells. Total proteins from blood samples were obtained using a whole blood protein extraction kit (Baiaoleibo, Beijing, China). After measuring protein concentration using the Pierce BCA Protein Assay Kit (Invitrogen, CA, USA), SDS-PAGE (ThermoFisher Scientific, CA, USA) was used to separate proteins. After constant pressure electrophoresis, proteins were transferred to the Immobilon-E PVDF membrane (Merck, Darmstadt, Germany). After cleaning the membrane with PBS three times, the membrane was incubated with BSA solution for 2 h at room temperature, and incubated with the primary antibodies at 4°C. Then, the goat anti-rabbit IgG H&L (HRP) was incubated with the membrane for 2 h at room temperature. Following washing with PBST, the blot was visualized using the Enhanced ECL Chemiluminescent Substrate kit (Yeasen Biotechnology, Shanghai, China).

The antibodies used in this study include anti-CPT1A (1:1,500, ab220789; Abcam, Cambridge, UK), anti-KAT2A (1:1,200, ab217876; Abcam), anti-KAT3B (1:2,000, ab275378; Abcam), anti-SIRT5 (1:1,000, ab259967; Abcam), anti-SIRT7 (1:2,000, ab259968; Abcam), anti-GLDC (1:1,000, ab204087; Abcam), anti-succinyllysine rabbit pAb (1:1,000, PTM-401; PTM Biolabs), anti-GADPH (1:3,000, ab9485; Abcam), and goat anti-rabbit IgG H&L (HRP) (1:2,000, ab205718; Abcam).

For GLDC protein stability determination, SIRT5 overexpressed AML cells were treated with 100 μg/mL cycloheximide. The protein levels of GLDC were detected by western blot at 0, 8, 16, 24 h, respectively.

### 2.6 Immunofluorescence (IF) staining

Cells were washed with PBS, immobilized with 4% paraformaldehyde for 20 min, permeabilized in 0.5% triton X-100 (Beyotime, Shanghai, China), blocked with 5% BSA, followed by incubation with primary antibodies (anti-SIRT5 and anti-GLDC) overnight at 4°C. After incubation with goat anti-mouse IgG H&L (Alexa Fluor^®^ 488, ab150113; Abcam) (green) and goat anti-rabbit IgG H&L (Alexa Fluor^®^ 647, ab150077; Abcam) (red), 4-6-diamidino-2-phenylindole (DAPI; Beyotime, Shanghai, China) (blue) was applied to stain cell nuclei. Finally, images were obtained using confocal microscopy (Leica, Germany).

### 2.7 Apoptosis assay

The apoptosis rate was determined using a modified flow cytometric method as described previously [29]. Briefly, HL-60 and KG1a cells at a density of 1 \[\times ]\] 10^5^ cells/well were incubated at 37°C 1 day before apoptosis assay. The cells were centrifuged to remove the culture medium. After adding 500 μL binding buffer, 5 µL 7-AAD solution, and 5 µL annexin V-FITC solution (Liankebio, China) in sequence, the solution was gently mixed. Cells were placed under darkness for 0.5 h and apoptosis was detected using flow cytometry.

### 2.8 TUNEL staining

A one-step TUNEL kit (Beyotime, Shanghai, China) was used for TUNEL staining of AML cell lines. After the treatment, cells were incubated with fluorescein-dUTP (Invitrogen, CA, USA) to stain apoptotic cell nuclei and DAPI (5 mg/mL) to stain all cell nuclei at room temperature for 3 min. The slides were imaged under a confocal microscope at least five random separate fields.

### 2.9 Cell counting kit-8 (CCK-8)

According to the manufacturer’s protocol of a CCK-8 kit (Beyotime, Shanghai, China), the transfected HL-60 and KG1a cell lines (3  ×  10^3^ cells/well) were treated with 10 μL CCK-8 solution for 2 h. The absorbance was detected using a microplate reader at 450 nm.

### 2.10 Co-immunoprecipitation (Co-IP)

The interaction between SIRT5 and GLDC was detected by Pierce’s classic magnetic bead IP/Co-IP kit (ThermoFisher, CA, USA) combined with western blotting. The cells were lysed with Co-IP lysis buffer, followed by incubating cell lysates and antibodies in test tubes overnight at 4°C to form immunoprecipitation complexes. The pre-treated protein A/G beads were then added to each tube and incubated again under ambient conditions for 1 h. Finally, a western blot was carried out using the supernatant containing the target antigen collected by magnetic scaffold.

### 2.11 Statistical analyses

Data were represented as mean ± standard deviation. Statistical differences between two and among multiple groups were calculated using Student’s *t*-test and one-way ANOVA, respectively. *p* < 0.05 was statistically different.

## 3 Results

### 3.1 SIRT5 expression is upregulated in patients with AML

**Figure 1:**
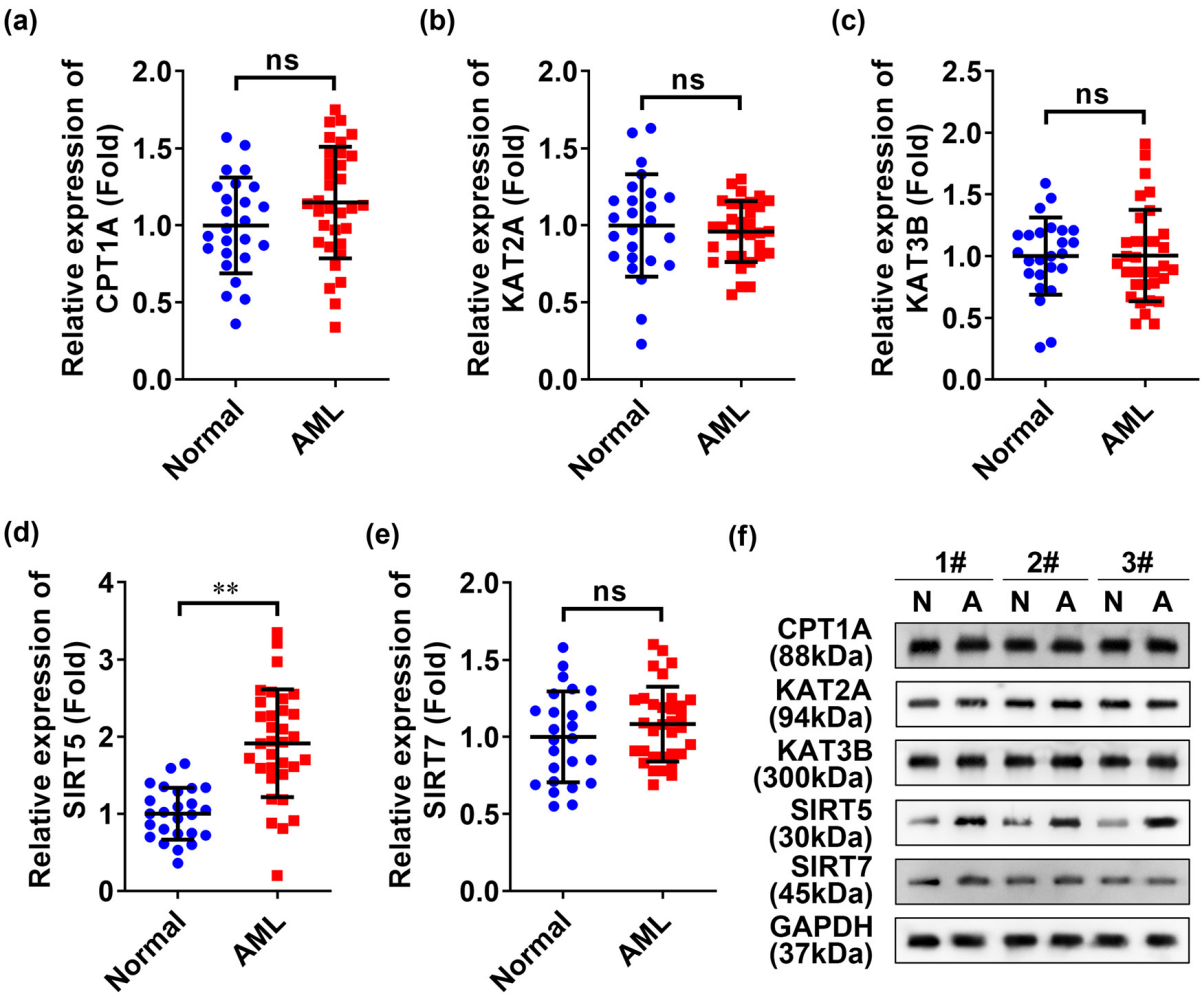
SIRT5 was highly expressed in clinical samples of AML. (a–e) mRNA expression levels of CPT1A, KAT2A, KAT3B, SIRT5, and SIRT7 in clinical samples of AML were detected by qRT-PCR. (f) Protein expression levels of CPT1A, KAT2A, KAT3B, SIRT5, and SIRT7 in clinical samples of AML were detected by western blot and the exemplary western blot images are shown (three samples). **p < 0.01; ns: no significant difference.

The expressions of succinyl transferases (KAT2A, KAT3B, and CPT1A) and desuccinylates (SIRT5 and SIRT7) for exploring succinylation modification effects on AML patients in blood samples of all participants. The qRT-PCR results showed that the mRNA expressions of KAT2A, KAT3B, CPT1A, and SIRT7 in blood samples of AML patients and healthy volunteers were insignificantly different (Figure 1a–c and e). Nevertheless, the mRNA expression of SIRT5 was significantly elevated in AML clinical samples (Figure 1d) compared to normal individuals, which was also similarly found in western blotting results (Figure 1f), indicating that the SIRT5 levels were significantly higher in AML patients; hence, it was speculated that SIRT5 was a key regulator during AML development.

### 3.2 SIRT5 knockdown suppresses viability and facilitates apoptosis of AML cells

**Figure 2:**
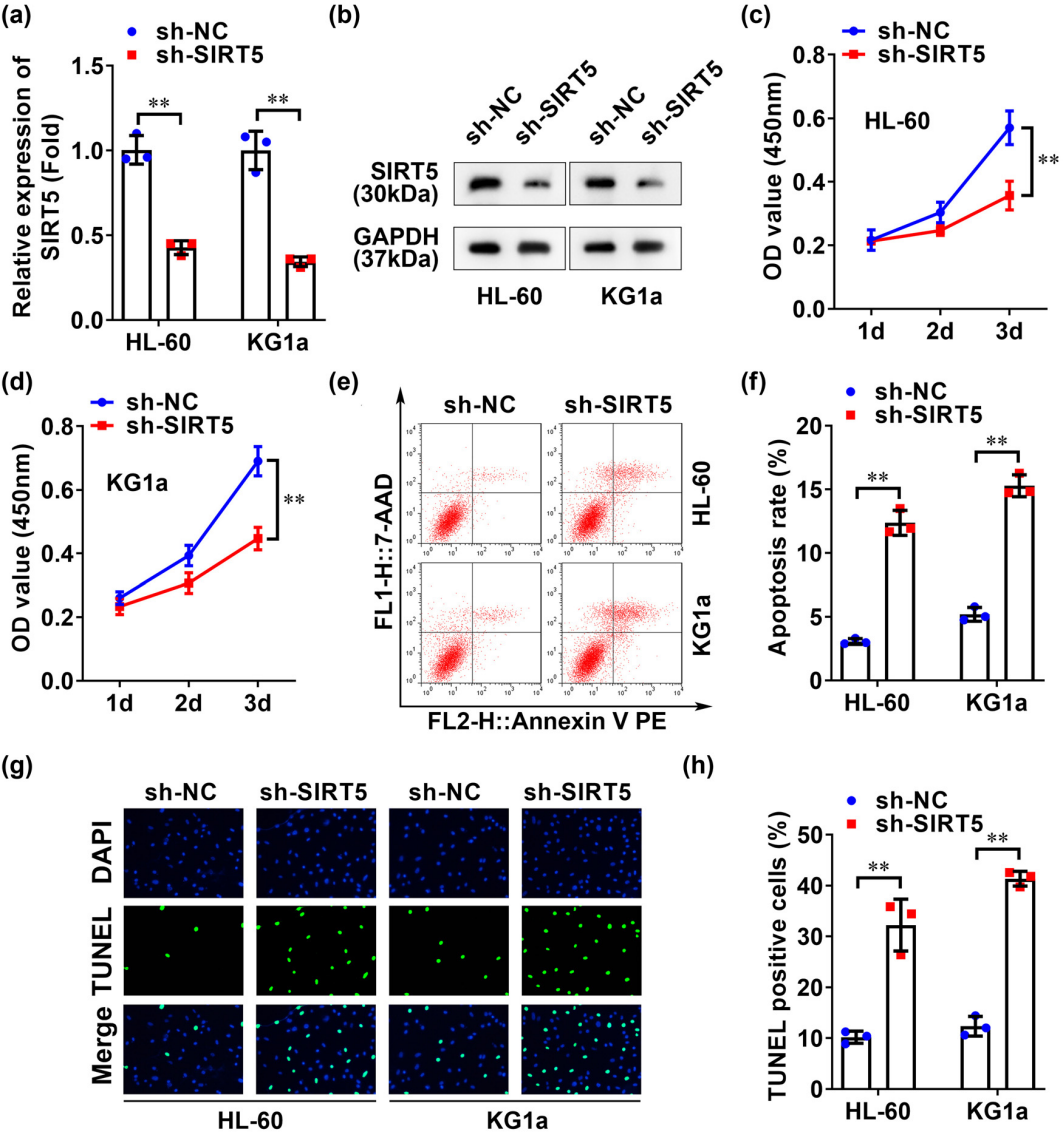
SIRT5 knockdown inhibited the viability and promoted apoptosis of AML cell lines. (a) qRT-PCR and (b) western blotting were used to detect the transfection efficiency of sh-SIRT5 in HL-60 and KG1a cell lines. CCK-8 detected the activity of (c) HL-60 and (d) KG1a cell lines. The apoptosis of HL-60 and KG1a cell lines was detected by flow cytometry (e and f) and TUNEL staining (g and h). **p < 0.01.

To confirm the role of SIRT5 in AML cells, the SIRT5 expression in AML cells was knocked down, and results showed that mRNA expressions of SIRT5 in HL-60 and KG1a cell lines after sh-SIRT5 transfection were significantly reduced (Figure 2a), in addition to considerably decreased protein levels (Figure 2b). These results indicated the successful transfection of sh-SIRT5. It was then proceeded by examining the post-sh-SIRT5 transfection cellular functions in AML cell lines, and results are shown in Figure 2c and d, indicating a significant inhibition of cell viability in both cell lines. Moreover, flow cytometric analysis (Figure 2e and f) and TUNEL staining (Figure 2g and h) revealed significantly elevated rates of apoptosis in both cell lines following SIRT5 knockdown.

### 3.3 SIRT5 modifies GLDC succinylation

**Figure 3:**
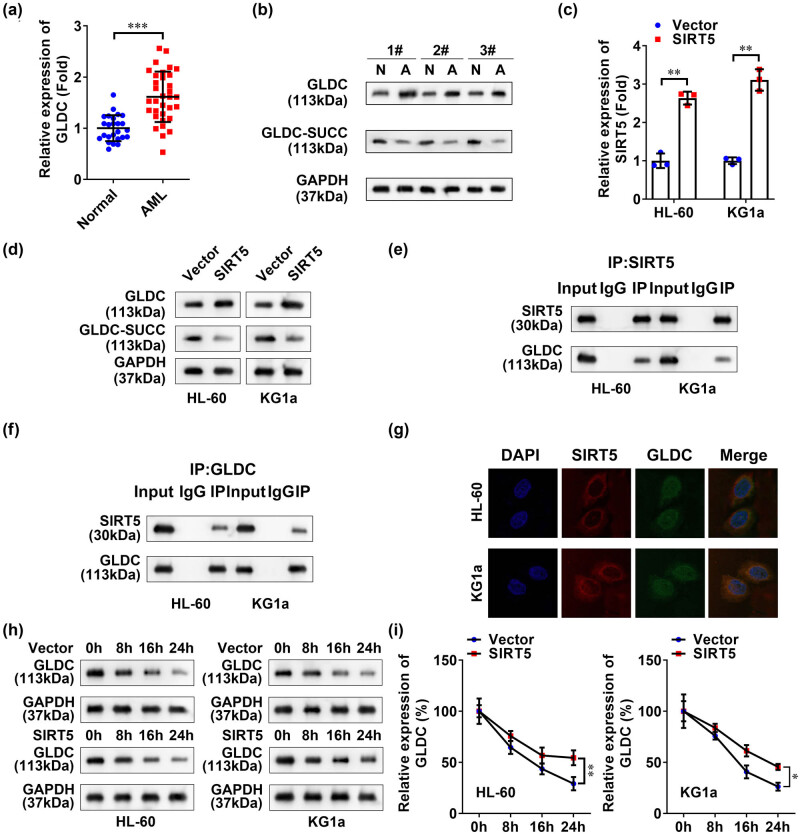
SIRT5 modified GLDC by succinylation. (a) mRNA levels of GLDC in clinical samples of AML were detected by qRT-PCR. (b) Protein expression and succinylation levels of GLDC in clinical samples of AML were detected by western blot and the exemplary western blot images are shown (three samples). (c) Overexpression efficiency of SIRT5 was detected by RT-qPCR. (d) Protein expression levels of GLDC and GLDC-SUCC after SIRT5 overexpression were detected by western blot. (e and f) Co-IP and western blot analysis were used to evaluate the interaction between GLDC and SIRT5. (g) IF staining was used to evaluate the binding between SIRT5 and GLDC. (h and i) Protein stability of GLDC in the SIRT5 overexpressed AML cell lines was detected by western blot. ***p < 0.001.

To elucidate the mechanism of SIRT5-mediated viability and apoptosis, GLDC levels regulated by SIRT5 were investigated. As shown in Figure 3a, GLDC mRNA levels were increased in the AML patients. In addition, the protein levels of FLDC were increased, while the succinylation levels of GLDC were decreased in the AML patients (Figure 3b). After SIRT5 overexpressed vector transfection, the SIRT5 mRNA levels were significantly increased in AML cell lines (Figure 3c). GLDC protein levels were increased when SIRT5 was overexpressed in AML cell lines, while the succinylation levels of GLDC (GLDC-SUCC) were decreased, suggesting that SIRT5 overexpression affected GLDC succinylation (Figure 3d). Moreover, analyzing binding patterns using Co-IP analysis of SIRT5 and GLDC (Figure 3e and f) showed that SIRT5 interacted with GLDC in both cell lines. Similarly, IF staining showed SIRT5 was co-located with GLDC within the nucleus (Figure 3g), which further cemented the speculation of SIRT5 and GLDC interaction. Furthermore, we found that SIRT5 overexpression inhibited the GLDC protein degradation in the AML cell lines, indicating SIRT5 enhanced the protein stability of GLDC (Figure 3h and i). All these results demonstrated that SIRT5 was involved in promoting GLDC desuccinylation in AML cells.

### 3.4 SIRT5 regulates GLDC-mediated viability and apoptosis of AML cells

**Figure 4:**
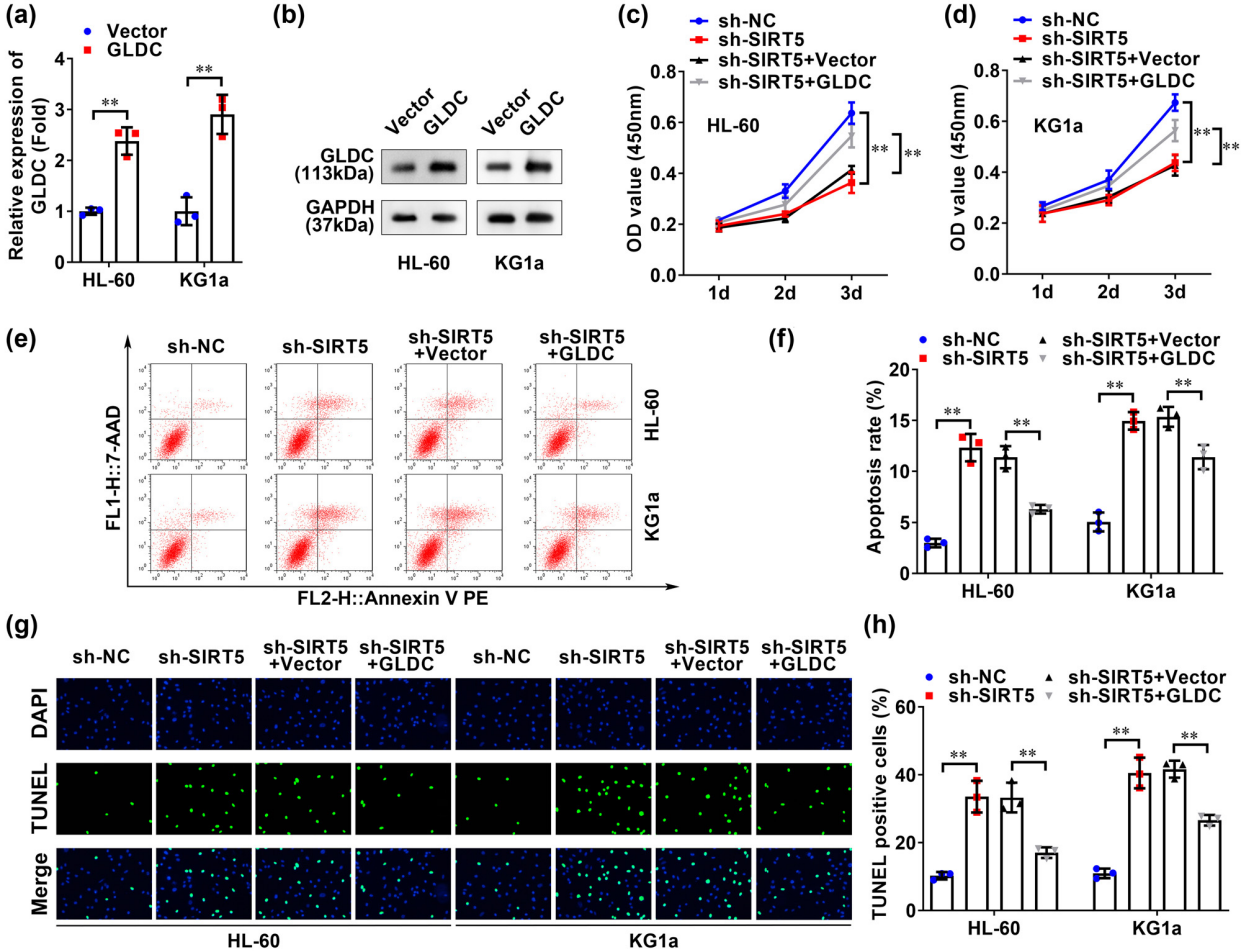
SIRT5 regulated AML cell viability and apoptosis by regulating GLDC. (a) qRT-PCR and (b) western blotting were used to detect the transfection efficiency of GLDC in HL-60 and KG1a cell lines. CCK-8 assays were used to detect the activity of (c) HL-60 and (d) KG1a cell lines. The apoptosis of HL-60 and KG1a cell lines was detected by flow cytometry (e and f) and TUNEL staining (g and h). **p < 0.01.

To further explore whether GLDC affects AML cellular processes, we analyzed the functional changes of HL-60 and KG1a cell lines after GLDC overexpression, and the results are shown in Figure 4a and b. The results demonstrated that GLDC levels were significantly increased in AML cell lines after GLDC transfection, cementing the successful transfection of GLDC overexpression plasmid into AML cell lines. As shown in Figure 4c and d, GLDC overexpression promoted cell viability in both cell lines, which was inhibited by SIRT5 knockdown. Additionally, flow cytometric analysis (Figure 4e and f) and TUNEL staining (Figure 4g and h) showed that GLDC overexpression inhibited apoptosis in both cell lines, which was promoted by SIRT5 knockdown. These results demonstrated that GLDC promoted AML cell viability and inhibited apoptosis, which was regulated by SIRT5.

## 4 Discussions

The process of succinylation has gained immense attention from researchers owing to its regulatory effect on various metabolic processes [30]. Our study aimed to analyze the specific regulatory mechanisms of desuccinylates and SIRT5 in the development of AML, and results showed that SIRT5 was involved in regulating AML cell viability and apoptosis by regulating GLDC succinylation modification.

AML is a fatal proliferative cancer caused by infinite clonal proliferation and differentiation loss of myeloid hematopoietic stem cells [31] with increasing incidence and mortality rates [32]. However, potential clinical targets for AML treatment have not been fully identified. Recently, succinylation has been shown to participate in many tumor cell processes, such as proliferation and apoptosis [33]. It was reported that the succinyl dehydrogenase complex regulated the development of AML by inhibiting the respiratory metabolism of AML cells [34], which was also confirmed in another study depicting that succinylation modifies the AML development [35]. Our results showed that desuccinylate SIRT5 expression was elevated in AML clinical samples, consistent with the study of Yan et al. [23], hence it was suggested that SIRT5 serves as a key regulator mediating AML development.

SIRT5 is the only known mitochondrial desuccinylates, belonging to the evolutionarily conservative sirtuin family [36], and is highly expressed in breast tumors, melanoma, and renal cell carcinoma [17,20,37,38]. Moreover, acetylation modification of SIRT6 in the same family has been proven to be a good target for leukemia therapy [39]. Similarly, NRD167, an inhibitor of SIRT5, has also been confirmed to improve the clinical outcomes of AML patients [40]. In our study, we found that knocking down SIRT5 in AML cell lines impeded cell viability and promoted apoptosis which was consistent with its functions in gastric cancer cells as reported earlier [41]. Moreover, our results showed that SIRT5 interacted with GLDC, a key enzyme regulating glycine, amino acid metabolism, and decomposing glycine into single carbon units, mediated its succinylation in AML cells [42]. It was reported that mTORC1 signaling was involved in inducing GLDC acetylation by SIRT3 in different tumors [43]. Our findings revealed for the first time that SIRT5 regulated GLDC succinylation.

GLDC expression is usually upregulated in tumor patients, playing a critical role in tumorigenesis and cancer progression, such as in breast and lung cancers, hepatocellular carcinoma, and leukemia [44–46]. It was revealed by Liu et al. that high GLDC expression inhibited stem cell injury in chronic myeloid leukemia [47], and was aberrantly expressed in AML clinical samples [42]. Our results of rescue experiments revealed that GLDC overexpression counteracted AMI cell viability and apoptosis regulated by SIRT5 knockdown. Combined with SIRT5 and GLDC interaction, it is suggested that SIRT5 was involved in mediating AML progression through the GLDC succinylation.
